# OSM-SLAM: Aiding SLAM with OpenStreetMaps priors

**DOI:** 10.3389/frobt.2023.1064934

**Published:** 2023-03-29

**Authors:** Matteo Frosi, Veronica Gobbi, Matteo Matteucci

**Affiliations:** Dipartimento di Elettronica, Informazione e Bioingegneria of Politecnico di Milano, Milan, Italy

**Keywords:** SLAM, OpenStreetMap, localization, LiDAR sensor, mapping

## Abstract

In the last decades, Simultaneous Localization and Mapping (SLAM) proved to be a fundamental topic in the field of robotics, due to the many applications, ranging from autonomous driving to 3D reconstruction. Many systems have been proposed in literature exploiting a heterogeneous variety of sensors. State-of-the-art methods build their own map from scratch, using only data coming from the equipment of the robot, and not exploiting possible reconstructions of the environment. Moreover, temporary loss of data proves to be a challenge for SLAM systems, as it demands efficient re-localization to continue the localization process. In this paper, we present a SLAM system that exploits additional information coming from mapping services like OpenStreetMaps, hence the name OSM-SLAM, to face these issues. We extend an existing LiDAR-based Graph SLAM system, ART-SLAM, making it able to integrate the 2D geometry of buildings in the trajectory estimation process, by matching a prior OpenStreetMaps map with a single LiDAR scan. Each estimated pose of the robot is then associated with all buildings surrounding it. This association allows to improve localization accuracy, but also to adjust possible mistakes in the prior map. The pose estimates coming from SLAM are then jointly optimized with the constraints associated with the various OSM buildings, which can assume one of the following types: Buildings are always fixed (Prior SLAM); buildings surrounding a robot are movable in chunks, for every scan (Rigid SLAM); and every single building is free to move independently from the others (Non-rigid SLAM). Lastly, OSM maps can also be used to re-localize the robot when sensor data is lost. We compare the accuracy of the proposed system with existing methods for LiDAR-based SLAM, including the baseline, also providing a visual inspection of the results. The comparison is made by evaluating the estimated trajectory displacement using the KITTI odometry dataset. Moreover, the experimental campaign, along with an ablation study on the re-localization capabilities of the proposed system and its accuracy in loop detection-denied scenarios, allow a discussion about how the quality of prior maps influences the SLAM procedure, which may lead to worse estimates than the baseline.

## 1 Introduction

Autonomous robot navigation has been an increasingly studied topic in recent decades. Many tasks, which are repetitive or hazardous to humans, may be carried out by means of unmanned agents. Examples include mobile robots for cleaning, agriculture and farming, automated driving vehicles, and robots for delivery and transportation on the road. These robots need to be built in a way that allows them to operate, safely and reliably, in complex or unknown environments, based only on the perceptions of their onboard sensors. To achieve this, an accurate map of the environment is mandatory, as it could be used for both planning and localization, with the latter being of particular interest in the field of robotics.

Simultaneous Localization and Mapping (SLAM) methods address the problem of constructing a model of the environment surrounding the robot, i.e., the map, while simultaneously estimating its pose within it. In literature, many SLAM systems can be found, relying either on cameras or laser range scanners (LiDARs) as main sensors. In this work, we focus on the latter, as LiDAR-based SLAM systems have been proven to be more robust w.r.t. Other approaches, mainly because of the way pose estimation and tracking are achieved (usually by scan-to-scan matching between consecutive point clouds, using well known algorithms). State-of-the-art systems can build a map of the environment using only equipped LiDARs and do not exploit existing prior maps, which can be integrated to achieve better localization. Moreover, temporary loss of input data, e.g., caused by a sudden sensor failure, proves to be a challenge for almost all methods, as it requires efficient re-localization, which is inherently difficult when considering point clouds, to continue the whole trajectory estimation and mapping process.

To address these problems, in this paper we propose an extension of ART-SLAM Frosi and Matteucci (2022), using it as the baseline for our system, in which we include information from OpenStreetMaps maps, hence the name OSM-SLAM. We contribute to the literature in the following ways:

• We improve the pose graph construction of ART-SLAM, achieved through the g2o framework Grisetti et al. (2011), adding knowledge about the buildings surrounding the robot, at a given position.
• We propose a SLAM system able to cope with three different scenarios. (1) Buildings are fixed (e.g., because they are assumed to be correct in the OpenStreetMaps map), and only the estimated trajectory of the robot is corrected (*Prior SLAM*). (2) Buildings surrounding a pose of the robot are not fixed, and they are optimized and moved by the same rigid motion, while simultaneously correcting the estimated trajectory (*Rigid SLAM*). (3) Lastly, buildings are again not fixed, but each entity is roto-translated w.r.t. A single input scan while constraining the corresponding robot pose (*Non-rigid SLAM*).
• We perform a quantitative and qualitative evaluation of the presented methods on sequences of the state-of-the-art dataset KITTI Geiger et al. (2012). Moreover, through an ablation study, we test the re-localization capabilities of our system (e.g., in case of sensor failure) and its behavior when loop detection and closure are not available (i.e., when only odometry and mapping are performed).

Our work stands from other approaches leveraging on external maps as we add buildings to the pose graph, which represents 3D information, as 2D nodes, actively participating in the optimization phase. This allows for simultaneously correcting both the estimated trajectory, obtained through full point cloud-based LiDAR tracking, and the buildings themselves, in case their placement in the corresponding OSM map is not coherent with at least one input scan. It should also be noted that, in our work, the pose graph is built using nodes belonging to different dimensions (2D for buildings and 3D for robot poses), while in the majority of works, all data are brought in the same dimension, usually 2D, as the OSM maps.

The rest of the paper is organized as follows, dealing with literature, proposed approach, and results:

• Section 2 describes the related works, to give a brief insight into existing systems for LiDAR-based SLAM and localization using OpenStreetMaps data (either full maps or only buildings).
• Section 3 explains the proposed system, OSM-SLAM, going into more detail about its pipeline and the three implemented scenarios (Prior SLAM, Rigid SLAM, and Non-rigid SLAM).
• Section 4 is dedicated to the experimental validation of the proposed system, which includes a visual evaluation and a discussion about the influence of the quality of the maps over the SLAM approach.
• Section 5 sums up and concludes the manuscript.

## 2 Related works

LiDAR-based SLAM methods produce very precise 3D scans of the environment, represented as sets of points (also known as point clouds), allowing for accurate localization of the robot and the construction of a dense map, differently from Visual SLAM algorithms. Although both methodologies are becoming increasingly accurate over the past years Cadena et al. (2016), laser-based systems are preferred over image-based methods, mainly because of their greater precision and ability to reconstruct the environment. The recent work in Garigipati et al. (2022) provides a comparison between algorithms of different types, showing how LiDAR SLAM outperforms Vision SLAM, especially in outdoor scenarios. Moreover, the authors of Zou et al. (2021) show how LiDAR-based systems can also be used in indoor environments, achieving high localization and mapping accuracy, even with reduced computational resources.

These systems can be classified as feature-based and full point cloud-based, depending on the way tracking and localization are performed. In feature-based approaches, 3D features are extracted from LiDAR scans, such as edges or planes, which are later matched to perform tracking. A well known system in literature, of this type, is LOAM Zhang and Singh (2014), a low-drift and real-time LiDAR odometry and mapping method. LOAM performs feature-to-edge and feature-to-plane scan matching to find correspondences between features extracted from a pair of input scans. As no form of loop closure is done, LOAM, although fast, quickly drifts along the estimated trajectory. To address this issue, Shan et al. proposed LeGO-LOAM Shan and Englot (2018), a revised and improved version of LOAM, consisting of five independent modules: segmentation, feature extraction, LiDAR odometry, LiDAR mapping, and transform integration. The same authors also proposed LIO-SAM (Shan et al., 2020), which still has LOAM as the baseline system, which couples mandatory IMU data and LiDAR scans to achieve fast results.

Although incredibly fast, feature-based methods are, in general, less accurate than full point cloud-based approaches. These kinds of systems perform scan matching between two whole LiDAR scans to find the 3D transformation which best represents the motion of the robot, aligning the two point clouds. Indeed, this is more computationally demanding than feature-based approaches, although it leads to very accurate results. To address this issue, a family of algorithms that efficiently solve the SLAM problem, based on non-linear sparse optimization, has been presented over the last decade, consisting of graph-based approaches, also known as Graph SLAM systems. These are based on the representation of the SLAM problem as a graph, named pose graph, where nodes represent entities such as poses of the robot, landmarks, and other elements of interest, and where edges represent constraints between the nodes. As Grisetti et al. describe in Grisetti et al. (2010), Graph SLAM approaches present many advantages over other methods, such as the possibility to model complex scenarios and the availability of different frameworks for efficient sparse graph optimization, to increase the overall performance of the localization and mapping procedures.

HDL Graph SLAM (Koide et al., 2018) is a full point cloud-based Graph SLAM system, the pipeline of which can be summarized in four steps: input scans pre-processing, scan-to-scan point cloud matching, ground detection, and pose graph building and optimization. Although accurate, the system is slow, especially when dealing with large point clouds. ART-SLAM (Frosi and Matteucci, 2022) enhances HDL, by adding performance improvements, efficient multi-step loop detection and closure, and a re-engineered structure, making it easy to customize and extend, while also being a memory-friendly zero-copy approach.

To further improve the accuracy in SLAM systems, over the past years, many works have been proposed, which exploit already available pre-computed 2D maps, coming from external mapping services, such as Google Maps, OpenStreetMaps (Haklay and Weber, 2008) (OSM), or maps from the local land registry. In particular, information from OSM seems to be the best choice, in terms of availability, as it requires no permissions or tokens to retrieve 2D data. Many systems relying on OSM include maps into the observation model of a Monte Carlo localization, as in Hentschel et al. (2008), in which buildings are extracted from the 2D map as a set of lines and are used to compute the expected range measurement at a given position of the robot, or as in Floros et al. (2013), where the trajectory of the robot is aligned w.r.t. The road network.

A more recent Graph SLAM system, proposed by Vysotska and Stachniss (2016), directly related LiDAR measurements with the data associated with buildings coming from OSM. This alignment is included in the pose graph, in form of a localization error w.r.t. The available OSM map. A disadvantage of this approach is that a precise alignment between buildings and LiDAR scans is required in order to get good accuracy on the estimated poses of the robot. However, alignment becomes difficult when there is a lot of clutter in the environment, typical of urban areas, or in zones when there are few buildings surrounding the robot (in the OSM map, not necessarily in the physical world). Other problems arise when buildings are not correctly positioned in OSM, caused by human errors when creating the 2D maps. Lastly, the OSM map could also not be representative of the environment, as the topology may change over time.

Instead of directly using OSM maps, Naik et al. (2019), proposed a graph-based semantic mapping approach for indoor robotic applications, which extends OSM with robotic-specific, semantic, topological, and geometrical information. They introduced models for basic structures, such as walls, doors, or corridors, which are semantically grouped into a graph. Its hierarchical structure is then exploited to allow accurate navigation, compatible with grid-based motion planning algorithms.

OpenStreetMaps provides, in general, many advantages, such as global consistency, a heavy-less map construction process, and a wide variety of publicly available road information. In early OSM-based works, the authors focused on improving localization methods by sensor-based perception Ort et al. (2018), without considering possible OSM inaccuracies, leading to trajectories suffering from local deviations. In Suger and Burgard (2017), the authors corrected the OSM global trajectory by matching it to the previously segmented road, using a LiDAR sensor, although they did not achieve robust results (because the correction depends strongly on the road segmentation). Similarly, the work in Li et al. (2021) used a similar approach, by correcting the OSM path using a cost map, built using combined camera and LiDAR data.

The work in Muñoz-Bañón et al. (2022) presented an autonomous navigation pipeline that exploits OSM information as environment representation for global planning. To overcome one major issue of OSM maps, i.e., low local accuracy, the authors propose a LiDAR-based Naive-Valley-Path method, which exploits the idea of valley areas to infer the local path always further from obstacles. This allows navigation through the center of trafficable areas, following the shape of the road, independently of OSM errors.

Lastly, Cho et al. (2022) proposed a vehicle localization (not SLAM) method purely based on OSM maps. Their method generates OSM descriptors by calculating the distances to buildings from a location in OpenStreetMap at a regular angle, and LiDAR descriptors by calculating the shortest distances to building points from the current location at a regular angle. Comparing the OSM descriptors and LiDAR descriptors yields a highly accurate vehicle localization result. Compared to methods that use prior LiDAR maps, the algorithm presents two main advantages: vehicle localization is not limited to only places with previously acquired LiDAR maps, and the method is comparable to LiDAR map-based approaches.

Following the ideas of Vysotska and Stachniss (2016) and Cho et al. (2022), in the following we introduce OSM-SLAM and show how the issues currently present in state-of-the-art approaches leveraging OSM information have been faced, providing a robust yet flexible system for 3D LiDAR SLAM in urban cluttered environments. Besides handling clutter and possible errors in the OSM information, our system is also able to perform re-localization using OSM maps as prior 2D maps, handling possible situations where sensor data is lost for brief time periods (e.g., sensor failure), and also substitute loop detection and closure.

## 3 OSM-SLAM

**FIGURE 1:**
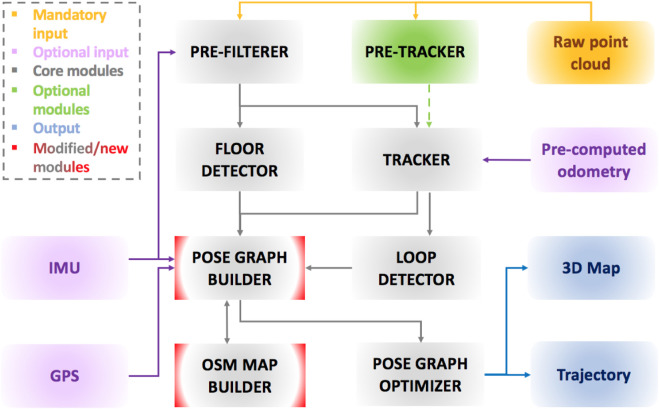
Architecture of the proposed system. The majority of core modules come from ART-SLAM Frosi and Matteucci (2022), while our improvements can be found in the pose graph builder, and in the new module *OSM MAP BUILDER*, which handles OpenStreetMaps data.

An overview of the proposed framework is presented in Figure 1. As already stated, our system is built upon an existing method, ART-SLAM Frosi and Matteucci (2022), with which it shares the majority of modules used to perform LiDAR-based SLAM. An input laser scan is processed to remove noise, reduce its size, and possibly deskew it. The new point cloud is then used to perform scan-to-scan matching, to estimate the robot motion, and possibly detect the ground plane, within the cloud, to later integrate it in the pose graph as height and rotational constraints. As for ART-SLAM, being a keyframe-based method, the proposed system extracts, from the tracker module, only a few odometry estimates (depending on the current rotation, translation, and time gap w.r.t. Previous scans). These estimates, along with corresponding point clouds, form a keyframe and are used to find loops in the trajectory and to build the pose graph.

**FIGURE 2:**
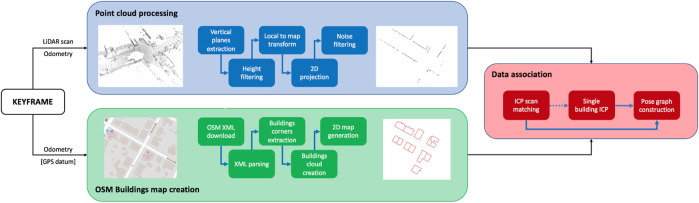
High-level pipeline describing the contribution of the proposed system. The whole procedure happens during the pose graph construction, before optimization takes place.

Before the optimization of the pose graph is performed, we introduce a new pipeline, to allow a direct association between point clouds and buildings derived from OSM data. The idea behind this pipeline is that by aligning a prior map with a LiDAR scan, one can obtain meaningful information about the pose of the robot (as the LiDAR scan is associated with it), w.r.t. The features present in the map (e.g., global coordinates of elements within it, like buildings), and *vice versa*. A high-level scheme of the approach is represented in Figure 2. Once a keyframe, obtained from the front-end of the SLAM system, is inserted in the pose graph, it is ready to be given as input to the whole OSM data association procedure.

Odometry and 3D point cloud related to the keyframe considered are processed to extract a 2D map of the buildings, while odometry and optional GPS data (only one datum in the whole trajectory is required to make the method work) are used to download and parse OSM data, to be converted in a 2D point cloud representing the buildings surrounding the robot. Once the buildings’ map and the map from the LiDAR scan are retrieved, ICP (Besl and McKay, 1992) scan matching is performed, to obtain a first rough estimate of the rigid motion that should move the robot towards its true position in the OSM map. This procedure is named *Rigid SLAM*, as scan matching is performed at a local level, involving all surrounding buildings.

Alignment can also (optionally) be repeated, using as an initial guess the just computed transformation, for each building, independently of one another. This allows for obtaining more precise information about the displacement of all buildings, and possible errors in the OSM map. As, following this approach, buildings now exist as separate entities w.r.t. The pose of the robot, differently from the previous case where the computed transformation was the same for all buildings surrounding the robot, the name given is *Non-rigid SLAM*. Independently from the selected approach, i.e., Rigid or Non-rigid, all the constraints of the type {robot pose, building} are added to the pose graph, ready for optimization.

It should also be noticed that buildings can be fixed during the graph optimization procedure, especially if it is known that the used 2D OSM map is precise and accurate. This solution, which takes the name of *Prior SLAM*, has the advantage of increasing the overall performance w.r.t. The non-fixed buildings counterparts, but it also maintains a relationship between the buildings and the pose of the robot, exploited in the optimization step. In other words, buildings are not modified but still constrain the estimated trajectory.

### 3.1 Point cloud processing branch

LiDAR scans cannot be directly aligned with a 2D prior map representing buildings, due to many issues. First, the point cloud is 3-dimensional, differently from the prior map, leading to convergence errors, assuming that the scan matching procedure even starts. Then, LiDAR scans represent a richer environment than the one given by OSM maps, including many elements of disturbance, such as vehicles, road signs, cyclists, fences, small walls, and even the ground itself. Lastly, using a whole point cloud would be wasteful, needing too many computational resources, due to the complexity of the scan matching algorithm.

To overcome these problems, we propose a small sequence of pre-processing operations, to be applied on an input point cloud, with the goal of obtaining a 2D map of the buildings, extracted from it (blue box of Figure 2). As a first step, we remove all non-vertical and non-plane-like elements of the cloud, as they should not be part of the final map. This is achieved through the iteration of a slightly modified version of RANSAC, i.e., Random Sample Consensus Model for Parallel Planes, to estimate all planes in the cloud which normal is perpendicular (meaning that the plane is parallel) to a given axis (we use the normal to the ground), within a threshold angle. In our experiments, we consider a maximum value of 10°, above which a plane is no longer considered a wall, to account also for errors in the LiDAR scans.

As a second step, we filter the obtained point cloud to only consider points above the LiDAR horizon. Indeed, it is very likely to find false walls between the ground and the considered height threshold, including small fences, cars, road signs, and so on. This way, we further de-clutter the point cloud, leaving in it almost only 3D points associated with walls and high vertical planes (e.g., panels or high vans).

We should remember that, up to now, the processed point cloud lives in the local coordinate frame, i.e., the one associated with the sensor. This approach is adopted to increase performance while performing iterated RANSAC for plane removal, which represents a quite expensive task. To perform accurate scan matching with a map derived from OSM, as a third step, we then transform the point cloud into the map coordinate frame, using the estimated corrected odometry (meaning that it follows a previous optimization, to take into account possible adjustments, e.g., a previous alignment with some OSM buildings).

Once transformed, the point cloud is projected from 3D to 2D, onto the ground plane *z* = 0. At this point in the pipeline, the cloud obtained is already usable to perform scan matching, having a similar representation to the OSM map. Nevertheless, we want to further clean up the 2D cloud, so, as a last step, we perform some noise filtering and outlier removal, using a simple radius search algorithm.

The blue box of Figure 2 follows the procedure described above, showing the initial point cloud to the left and the processed buildings map to the right. Thanks to the pre-processing, we are able to obtain great data reduction, while simultaneously refining more and more an initial scan to better fit our purposes. However, the iterative RANSAC proves to be a bottleneck in the pipeline, as it involves a continuous search in the initial cloud. We believe that this step can be interchanged with equivalent methods, which are beyond the scope of this paper, e.g., deep learning-based 3D point cloud segmentation or advanced clustering.

### 3.2 OSM buildings map creation branch

Data downloaded from third-party mapping services such as OpenStreetMaps do not come already in the form of point clouds. Instead, they are given in a specific data format and structure, typical of many systems. For this reason, it is mandatory to collect OSM data surrounding the robot position and process it, to create a suitable point cloud representation that allows fast and efficient scan-to-scan 2D matching.

OSM implements a conceptual data model of the physical world based on components called elements. There are three types of elements: nodes, ways, and relations. All elements can have one or more associated tags. A node represents a point on the surface of the Earth and it comprises at least an ID number and a pair of high-precision coordinates corresponding to the latitude and longitude of the point. A way is an ordered list of nodes that define a poly-line. It can be used to represent linear features, such as roads or rivers, or the boundaries of areas and polygons (closed way), like buildings or forests. A relation is a multi-purpose data structure that tells the relationship between two or more data elements, being an ordered list of nodes, ways, and other relations. Lastly, a tag describes the meaning of the particular element to which it is attached. It is made up of two fields: a key and a value, both represented as strings of characters. The key describes the meaning of the tag, such as “highway”, and it is unique. The value is the description of the key, such as “residential”, and it gives more detailed information, associating semantic value.

**FIGURE 3:**
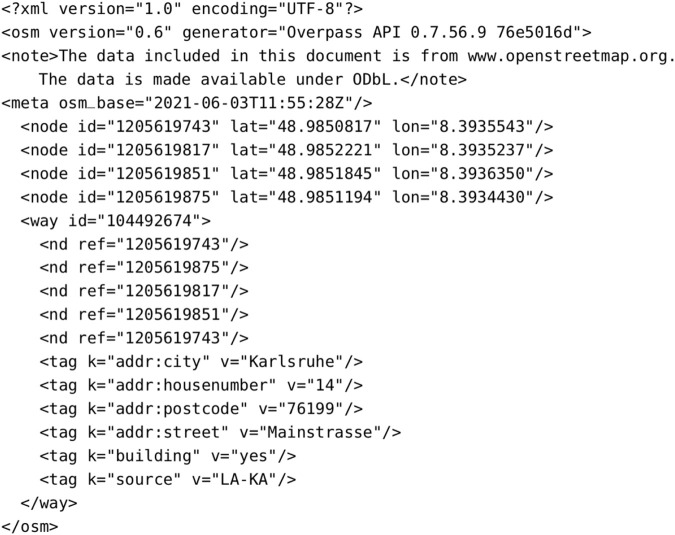
Example of an OSM XML response, following a query to the Overpass API. The structure described in this response is a building, as can be seen by its shape and tags.

It is possible to access and download map data from the OSM dataset in many ways. The most convenient, and suitable for the purpose of our work, is the Overpass API. It is a read-only API that allows accessing parts of the OSM map data selected in a custom way, given search location (latitude and longitude) and radius. It allows the client to send a query through an HTTP GET request to the API server, which will, in turn, send back the dataset that resulted from the query. There exist two languages in which to write a query: Overpass XML or Overpass QL. The Overpass QL syntax is more concise than Overpass XML and is similar to C-like programming languages. On the other hand, the Overpass XML syntax is safeguarded, because it uses more explicit named parameters than QL. We chose the QL language because it is easier to use. The response can also be in different formats, such as OSM XML, OSM JSON, custom templates, and pretty HTML output. In this case, we chose to get the data in the OSM XML format, as it can be parsed in an efficient and fast way, suitable for SLAM. Figure 3 shows an XML response, following a query to the Overpass API. Aside from tags of the header, other elements inside the response are nodes and ways, as described above. In particular, inside the way, *nd* elements are specified, whose attribute *ref* coincides with the id of a node belonging to it. As the tags suggest, the object represented by the way in the response is a building, as we are interested in such structures to later align them with LiDAR scans.

As a first step, to query the Overpass API and find the buildings surrounding the robot, we need a position to center the search of buildings. In fact, the query has the following structure: *(way[”building”](around: radius, latitude, longitude);); (._;* > *;); out;*, which retrieves all buildings in a certain area specified by a radius centered in a given position. As we have seen before, the geographic position in longitude and latitude coordinates is required. GNSS data is not always available, or even reliable, as it may happen in complex scenarios (due to errors caused by the Urban Canyon effect) or tunnels. For this reason, the position, estimated through tracking, associated with the considered keyframe is used as a geographic location to query the Overpass API and download buildings. The position used is the same as mentioned in Section 3.1 when converting the LiDAR scan from local to map coordinate frame. Nevertheless, at least one GNSS datum is required to give a rough estimate of where, in the world, the robot is located. This value serves as the origin of the map, and it is used to convert both estimated positions of the robot and buildings of OSM from Longitude-Latitude-Altitude to East-North-Up (ENU) coordinates and *vice versa*.

From this query, we are able to obtain a list of buildings surrounding the estimated pose, of the keyframe, in the format described above. This response is then parsed, to form a point cloud for each building, containing the 2D points representing its corners, referenced w.r.t. The GNSS origin (meaning that the corners are expressed in ENU coordinates, easier to work with). Given the corners, for each building, we interpolate its edges, generating 100 to 1000 points for edge. In this way, we obtain a structured cloud, fully representing the buildings. Lastly, all the point clouds associated with the various buildings are merged, to form an accurate 2D map of the buildings surrounding the robot. This map is now ready to be matched against the map computed in the point cloud processing branch, previously described in Section 3.1.

### 3.3 Data association - Rigid SLAM and prior SLAM

Following the procedures described in Section 3.1 and Section 3.2, the proposed system is able to obtain, starting from the pose associated with a keyframe and the corresponding point cloud, a rough 2D map that contains the visible edges of the buildings surrounding the robot and a refined 2D map formed by the contours of all buildings, derived from OSM data collected at the position of the robot.

These two point clouds are matched using the ICP algorithm, fine-tuned to obtain very accurate alignments. Despite ICP suffering from local minima, point clouds and buildings are more or less already aligned, leading to an almost certain convergence of the matching method. Moreover, other algorithms (e.g., Generalized ICP or NDT) did not lead to more accurate results, being, instead, more time-consuming.

**FIGURE 4:**
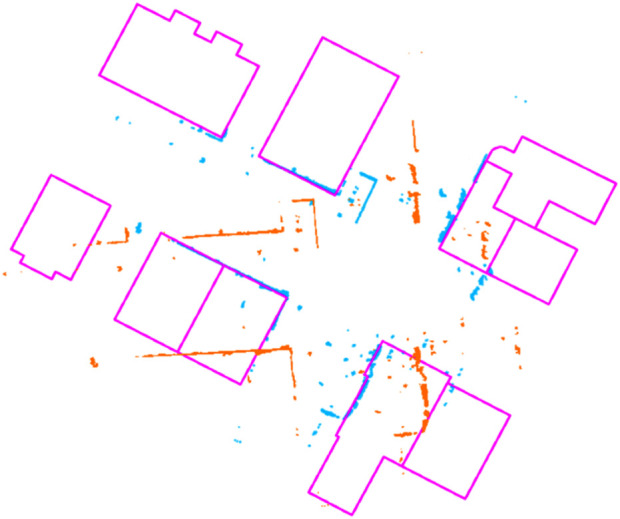
Example of alignment between the 2D buildings’ map from OSM (pink) and the 2D map extracted from LiDAR scan (orange). The aligned point cloud (blue) correctly overlaps the buildings’ map.

**FIGURE 5:**
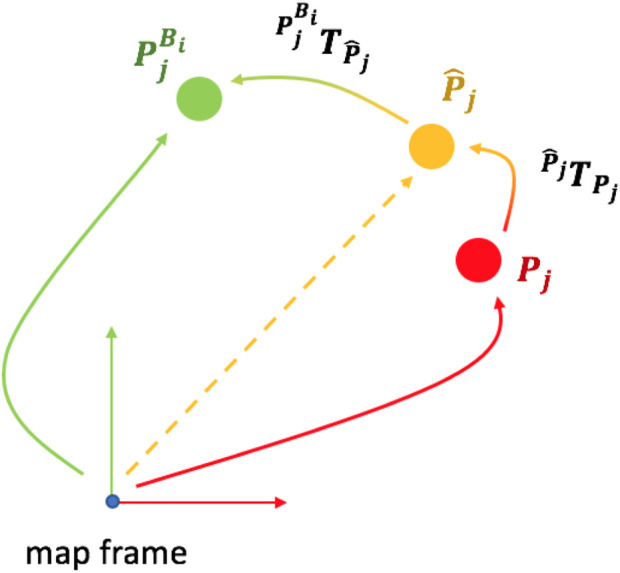
First, we find the correct position of the robot ${\hat{P}}_{j}$ , w.r.t. OSM buildings map. Then, we compute the displacement between ${\hat{P}}_{j}$ and the pose of the building, $P_{j}^{B_{i}}$ .

Figure 4 shows the result of this alignment: the 2D map extracted from the LiDAR scan (orange) is matched against the OSM map (pink), obtaining an accurate transformed point cloud (blue, clearly overlapping the pink one, as it should). Now that the transformation is computed, we need to add this constraint to the pose graph, along with odometry or other kinds of constraints. Figure 5 can be used to understand the insertion of new elements in the pose graph, including also buildings, described as follows.

Given a building *B*_*i*_, we always set its reference frame in the corner with the lowest *x* value, in ENU coordinates. $P_{j}^{B_{i}}$ is the 2D position of said corner, when associated with keyframe *j* (note that the same building can be seen by multiple keyframes, but it has only one associated position, i.e., the one that best satisfies all pose graph constraints). Building *B*_*i*_ is inserted in the pose graph as a node, which position corresponds to $P_{j}^{B_{i}}$ . In particular, the translation part is its ENU coordinates, while the rotation is the 2-by-2 identity matrix, meaning that the local frame of the building is oriented as the ENU coordinates.

Given the estimated position of the robot *P*_*j*_, again related to keyframe *j*, to which correspond a node in the pose graph, we would like to associate it with the building node *B*_*i*_, through some sort of measurement. Let ${\hat{P}}_{j}$ be the correct position of the robot w.r.t. The OSM map. Ideally, $P_{j}={\hat{P}}_{j}$ , which means that the current estimated position of the robot is also the correct position w.r.t. OSM. In practice, this does not happen, and the scan matching algorithm would return a rigid transformation between the buildings map and the processed LiDAR scan. We name this transformation TPjP^j , as it moves the estimated position towards the correct one, such that:

P^j=TPjP^j*Pj. (1)

Now that we have an adjusted value of the pose of the robot w.r.t. The buildings map of OSM, we are able to find the rigid motion between the corrected keyframe and the pose of the associated building *B*_*i*_, computed as:

TP^jPjBi=PjBi*P^j−1. (2)

This transformation suggests how rotated and translated are, w.r.t. Each other, the position of the building (corner), and the estimated pose of the robot. The constraint is inserted into the pose graph as an edge between the two corresponding nodes and is later used in the optimization phase to adjust and correct the positions associated with the nodes. To better understand this concept, one can think, again, at the ideal case, where $P_{j}={\hat{P}}_{j}$ . What happens in the pose graph is that nodes and constraints already satisfy an equality with no measurement error, as there is no displacement between the estimated and correct pose (same by assumption). However, this scenario does not happen, and the given measurement yields some information about the true displacement between the robot and the buildings, which is later satisfied and corrected by the optimizer. In other words, we try to align the LiDAR scan with the contour of the building.

The whole procedure takes the name of Rigid SLAM, as it always estimates a rigid motion between the considered keyframe and the buildings, considered as a whole, surrounding the associated robot pose. When inserted in the pose graph, all buildings are treated equally, as the same transformation TPjP^j is used to find the measurements needed to characterize the relative constraint, as previously described. Nevertheless, consecutive keyframes may lead to different transformations, making the Rigid SLAM approach only “rigid” for each keyframe independently. Moreover, one can decide to fix the position of the buildings, if it has prior knowledge that the OSM map is mostly correct. In this case, which takes the name of Prior SLAM, the poses associated with the corners of buildings are not modified during the optimization procedure. Nevertheless, their influence on the keyframe is considered in the same way as described for Rigid SLAM, as they are associated with it by transformation TP^jPjBi , for all buildings.

### 3.4 Data association—Non-rigid SLAM

As we have seen in Rigid SLAM, a single global alignment is performed to associate the pose of a keyframe and the corresponding map of the buildings surrounding it, downloaded from OSM. Although simple to implement and relatively accurate, this approach may lead to some problems. When multiple keyframes see the same building in almost the same location, even if the building is wrongly positioned w.r.t. The real world, its position would never be modified. In other words, the Rigid SLAM method is able to move and correct buildings, but is not able to tell whether a building is in a wrong or correct position in the real world, leading also to mistakes in the optimization procedure, e.g., incoherence between the relationships keyframe - buildings across multiple, consecutive, keyframes.

To address this issue, once the global alignment is computed, each building is taken individually, to repeat the same procedure described in Section 3.3, with few differences. Before, we tried to align the LiDAR scan, associated with the considered keyframe *j*, against the point cloud that represents all the contours of the building surrounding the robot at that precise location. This alignment resulted in transformation TP^jPjBi , which is the same for all buildings. In other words, let *i* ∈ {*k*, *k* + 1, *…*, *k* + *s*} be all the buildings in the OSM map surrounding the robot whose estimated position is described by keyframe *j*. In Rigid SLAM, we obtain the same transformation, such that:

TP^jPjBk=TP^jPjBk+1=⋯=TP^jPjBk+s−1=TP^jPjBk+s,i∈k,k+1,…,k+s (3)

In Non-rigid SLAM, after this procedure, each building is considered separately, being formed by a relatively low number of 2D points. Then, scan matching is performed between the LiDAR scan and the point cloud of the single building. This alignment is aided using transformation TP^jPjBi as an initial guess, to facilitate it and boost performance. The resulting motion T^P^jPjBi directly tells the correct position of the building w.r.t. The keyframe, and it is independent of the transformations derived from the other buildings.

Let us consider again buildings *i* ∈ {*k*, *k* + 1, *…*, *k* + *s*}, located around the robot w.r.t. Keyframe *j*. From Rigid SLAM, we are able to align the LiDAR scan of the keyframe with the point clouds formed by all 2D buildings, resulting in a global transformation TP^jPjBi . It should be noticed that this relative motion is the same for all entities since they are considered jointly. In Non-rigid SLAM, the buildings are then considered separately one from the other and are once again aligned with the point cloud of the same keyframe as before, using the global transformation as the initial guess. This way, for each building we can obtain a local transformation T^P^jPjBi , which is more precise and captures the true displacement between the 2D structure and the LiDAR scan, since it will be used later in the optimization step.

Differently from Rigid SLAM, each building is treated as a standalone element in the pose graph, each associated with at least one keyframe by a unique transformation. With Non-rigid SLAM, we are able to detect errors and incorrect information present in the OSM map by using LiDAR scans, which directly model the environment surrounding the robot. From a local alignment, in fact, we are able to check for discrepancies between the OSM map and the map built using SLAM, possibly caused by annotation errors or due to a large time gap between LiDAR acquisitions and the creation of the OpenStreetMaps outline.

## 4 Experiments

We compare the proposed system with different methods for LiDAR SLAM: LOAM (Zhang and Singh, 2014), LeGO-LOAM (Shan and Englot, 2018), LIO-SAM (Shan et al., 2020), HDL (Koide et al., 2018) and the baseline of our work, ART-SLAM (Frosi and Matteucci, 2022) (without Scan Context). In particular, we evaluate all three variants of OSM-SLAM. First, we consider Prior SLAM, which follows the same procedure as in Rigid SLAM (as described at the end of Section 3.3), in which the nodes in the pose graph associated with buildings are fixed and therefore cannot be modified by the optimization procedure. Then, we evaluate both Rigid and Non-rigid SLAM approaches, to see their differences and behaviors.

To conclude the results, we perform an ablation study of the system, considering two different scenarios. In the first study, we present a re-localization experiment on one of the sequences used for testing, where we stop the tracking at the beginning for about 100 consecutive frames, and use OSM buildings maps to estimate the missing odometry and evaluate the re-localization possibilities using external mapping services. In the second scenario, we disable loop closure and run Prior SLAM, comparing it with the baseline.

We evaluate all systems on Sequence 07 and Sequence 00 of the KITTI odometry dataset (Geiger et al., 2012), as they correspond to trajectories with medium and high complexities, respectively, and the associated OSM maps are sufficiently detailed for testing. Experiments are done on a 2021 XMG 64-bit laptop with Intel(R) Core(TM) i7-11800H CPU @ 2.30GHz x 8 cores, with 24,576 KB of cache size.

### 4.1 Comparison and results

The goodness of the methods used for the comparison is computed by means of absolute trajectory error (ATE). With this metric, we are able to measure the difference between the estimated and true poses of the robot. As some of the considered methods are all keyframe-based, meaning that only a fraction of the input scans is inserted in the pose graph, we perform a pre-processing step to filter out ground truth elements corresponding to the keyframes indices and timestamps. We also include here a detailed visual evaluation of the estimated trajectory and placement of the buildings in the global map, overlapped with OSM tiles.

**FIGURE 6:**
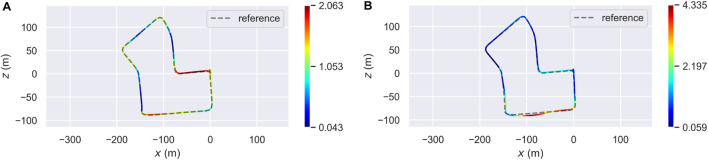
Localization accuracy of two proposed approaches (Prior SLAM on the left, Non-rigid SLAM on the right) on Sequence 07 of the KITTI odometry dataset Geiger et al. (2012). **(A)** Error distribution (Prior SLAM). **(B)** Error distribution (Non-rigid SLAM).

Table 1 details the obtained results in terms of mean, root mean square error (RMSE), and standard deviation (STD) of the ATE, in meters. Looking at the table, one may infer that the overall accuracy of the proposed system, in all three approaches, decreased w.r.t. The baseline, even if by an acceptable amount. However, using only these statistics does not explain in detail how the systems behave when dealing with OSM. Figure 6 shows the distribution of the ATE over the whole trajectory. In particular, Figure 6A refers to the results obtained with Prior SLAM, while Figure 6B represents the trajectory estimated with Non-rigid SLAM. It is clear how, especially for the latter case, the majority of the estimates prove to be definitely more accurate than the baseline, reaching error values below half a meter. Other sections, however, heavily influence the overall ATE mean value, and this is caused by the way buildings are placed in the corresponding high-error areas. For example, the segment of trajectory having the highest ATE is associated with a sequence of tightly joint buildings, meaning that the corresponding point clouds are just straight lines, tricking ICP into wrong alignments both in travel and orthogonal direction.

**TABLE 1:** ATE on Sequence 07 of the KITTI odometry dataset Geiger et al. (2012).

ATE [m]	MEAN	RMSE	Standard deviation
*LOAM*	> 10	> 10	> 10
*LeGO-LOAM*	1.191	1.309	0.546
*LIO-SAM*	0.509	0.675	0.351
*HDL*	0.954	1.253	0.767
*ART-SLAM (baseline)*	0.698	0.777	0.341
*Prior SLAM (ours)*	1.084	1.198	0.510
*Rigid SLAM (ours)*	3.253	3.420	1.057
*Non-rigid SLAM (ours)*	0.912	1.157	0.485

**FIGURE 7:**
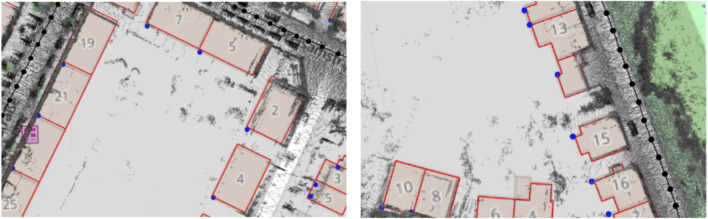
Detailed view of the reconstruction obtained with Non-rigid SLAM on Sequence 07. Notice the displacement of the buildings (blue dots) w.r.t. Their original location (red shape).

These results also give us more insight into the proposed systems. In case of Prior SLAM, where buildings are fixed, the constraints between buildings and keyframes are completely weighted on the keyframes themselves, leading to an overall distribution of the trajectory error. On the other hand, in Non-rigid SLAM, all buildings, independently one from the other, are free to move and adjust their location in the map, jointly with keyframes. This means that the trajectory error will be majorly concentrated in areas with possible issues. Lastly, one can also see the optimization effects on the buildings in Figure 7, which shows two detailed areas of the reconstruction with Non-rigid SLAM, overlapped to OSM tiles. Elements are slightly moved (blue dots), w.r.t. Their original position (red shape), to satisfy all constraints of Section 3.4.

**FIGURE 8:**
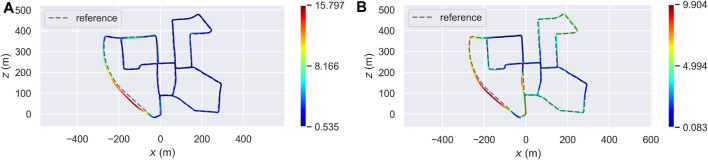
Localization accuracy of two proposed approaches (Prior SLAM on the left, Non-rigid SLAM on the right) on Sequence 07 of the KITTI odometry dataset Geiger et al. (2012). **(A)** Error distribution (Prior SLAM). **(B)** Error distribution (Non-rigid SLAM).

From there, we moved to Sequence 00 of the same dataset. Table 2 contains the ATE statistic. As in the previous case, the accuracy of OSM-SLAM seems to be worse than the baseline. Remembering the same reasoning done for Sequence 07, one should also look at the trajectory error distribution, to effectively understand the impact of OSM maps. Figure 8 represents the distribution of the ATE over the whole trajectory, with Figure 8A referring to the Prior SLAM case, and Figure 8B showing the results of Non-rigid SLAM. In the first case, most of the estimated trajectory has a low error, almost half of the baseline, whereas in a small area (curved road on the bottom right side, where only a few OSM buildings can be found) we see a noticeable drift that influences the mean error. On the other hand, in the Non-rigid SLAM scenario, we can see that the error is spread over the trajectory, reaching the lowest values of less than 10 cm (which is quite outstanding on such a complex map, containing multiple turns and loops).

**TABLE 2:** ATE on Sequence 00 of the KITTI odometry dataset Geiger et al. (2012).

ATE [m]	MEAN	RMSE	Standard deviation
*LOAM*	> 10	> 10	> 10
*LeGO-LOAM*	9.537	11.666	6.718
*LIO-SAM*	> 10	> 10	> 10
*HDL*	1.378	1.424	0.779
*ART-SLAM (baseline)*	0.981	1.092	0.478
*Prior SLAM (ours)*	3.802	4.778	2.893
*Rigid SLAM (ours)*	4.064	4.494	1.918
*Non-rigid SLAM (ours)*	3.648	4.214	2.110

**FIGURE 9:**
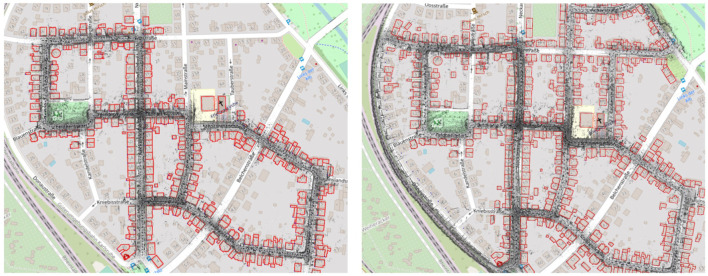
Evolution of the trajectory and 3D map estimation on Sequence 00, using Prior SLAM.

Lastly, we show the evolution of the 3D reconstruction and trajectory estimation (with Prior SLAM), visible in Figure 9. Already at half of the traversed path, visible in the left panel, the constructed map shows a high level of accuracy and details, which is kept for the rest of the whole SLAM procedure, and it ends in the right panel of the figure, which depicts the complete 3D reconstruction of Sequence 00.

### 4.2 Ablation study

**FIGURE 10:**
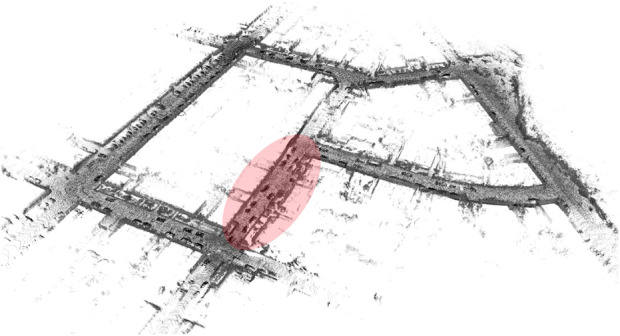
3D map obtained through the re-localization experiment. The red zone consists of frames that are not used for tracking, and for which the odometry is computed through OSM map alignment.

As the first experiment of the ablation study, we tried to evaluate the re-localization capabilities of OSM-SLAM on Sequence 07. This was achieved by simulating a faulty tracking, for about 100 consecutive frames (slightly less than 10% of the whole sequence), right at the beginning of the trajectory, as depicted in Figure 10. Then, we used the available OSM maps to re-localize the robot within it (using the same scan matching procedure described for both Rigid SLAM and Non-rigid SLAM, in Section 3.3; Section 3.4, respectively) automatically estimating the relative motion of consecutive LiDAR scans. While the baseline of the system would completely fail, OSM-SLAM works as intended, after re-localization, with a small loss in accuracy (a few centimeters worse than ART-SLAM). This proves that re-localization can be accurately performed through the means of 2D maps coming from mapping services, such as OpenStreetMaps.

In the second experiment of the ablation study, instead, we disabled the loop detection and closure module of both baseline and Prior SLAM, evaluating the ability of the proposed system to compensate for drift errors in the absence of loops. Table 3 shows the absolute trajectory error statistics (not just mean, RMSE, and standard deviation, as done in Section 4.1), of the two compared systems, both on Sequence 07 and Sequence 00. Prior SLAM definitely outperforms the baseline in every aspect when tested on the medium-length path (Sequence 07), which contains only one loop at the end. ART-SLAM, instead, is slightly more accurate, on average, on the other trajectory, Sequence 00, due to the fact that many buildings are missing in the map or are slightly misplaced w.r.t. The LiDAR measurements. It is noticeable, however, that Prior SLAM always shows the lowest minimum error and standard deviation, reaching values of about 4 cm for Sequence 07 and 6 cm for Sequence 00 (more accurate than when loop detection was enabled). This confirms what was stated in the previous section, i.e., that the proposed system (Prior SLAM, in this case) is more locally accurate than other methods and more stable over long runs.

**TABLE 3:** ATE statistics on Sequence 07 and Sequence 00 of the KITTI odometry dataset Geiger et al. (2012), evaluating Prior SLAM and the baseline with disabled loop detection and closure. Bold numbers represent the best values for each metric in the two sequences.

ATE [m]	Sequence 07		Sequence 00	
	Prior SLAM (ours)	ART-SLAM	Prior SLAM (ours)	ART-SLAM
*Max*	**2.670**	4.199	11.064	**9.820**
*Mean*	**1.166**	1.537	3.152	**2.978**
*Median*	**1.194**	1.368	3.129	**2.544**
*Min*	**0.044**	0.228	**0.064**	0.212
*RMSE*	**1.306**	1.799	3.838	**3.629**
*SSE*	**225.101**	427.399	8958.518	**8005.911**
*STD*	**0.587**	0.936	**1.869**	2.074

## 5 Conclusion

In this paper, we presented OSM-SLAM, a LiDAR-based Graph SLAM system that exploits OSM data to extract geometric information of buildings surrounding a robot and use this information to optimize the estimated trajectory, while simultaneously correcting possible mistakes in the corresponding OSM map. In particular, we presented three variants of the system, i.e., Prior, Rigid, and Non-Rigid SLAM, which respectively try to align a LiDAR scan with either a complete 2D map of fixed buildings from OSM, non-fixed buildings, or the contour of each structure, considered separately one from the other. We showed that the proposed systems achieve equal or better local accuracy than the baseline (also when loop detection is disabled), while also proving that re-localization using external mapping services, like OSM, is possible even in case of considerable data loss, without impacting much the estimated trajectory.

## Data Availability

Publicly available datasets were analyzed in this study. This data can be found here: https://www.cvlibs.net/datasets/kitti/eval_odometry.php.
